# Re-conceiving building design quality: A review of building users in their social context

**DOI:** 10.1177/1420326X14557550

**Published:** 2014-11-10

**Authors:** Kelly J. Watson, James Evans, Andrew Karvonen, Tim Whitley

**Affiliations:** 1 School of Environment, Education and Development, University of Manchester, UK; 2 NWY Building Engineering, Arup, UK

**Keywords:** Building user, Building user group dynamics, Design quality, Social context, Social value, Typology of buildings

## Abstract

Considerable overlap exists between post-occupancy research evaluating building design quality and the concept of ‘social value’, popularised by its recent application to issues of the public realm. To outline this potential research agenda, the paper reviews design quality research on buildings in relation to users and their social context where the term ‘social context’ refers to building user group dynamics, a combination of organisational cultures, management strategies, and social norms and practices. The review is conducted across five key building types, namely housing, workplaces, healthcare, education, and the retail/service sector. Research commonalities and gaps are identified in order to build a more comprehensive picture of the design quality literature and its handling of users in their social context. The key findings concerning each building type are presented visually. It is concluded that the design quality field comprises a patchwork of relatively isolated studies of various building types, with significant potential for theoretical and empirical development through interdisciplinary collaboration. Users tend to be conceived as anonymous and autonomous individuals with little analysis of user identity or interaction. Further, the contextual impact of user group dynamics on the relationship between building design and building user is rarely addressed in the literature. Producing a more nuanced understanding of users in situ is proposed as an important area for future design quality research.

## Introduction

### Design quality research

A significant proportion of the research that exists on buildings addresses post-occupancy issues, rather than design process, and how they relate to the quality and functioning of the building in use. The literature is split between studies whose primary goal is to gather data about building users in order to evaluate design quality^1^ and those that focus on assessing economic profit^2^ or environmental performance.^3^ A variety of approaches exist which identify user experience as the unit of analysis but they vary considerably according to their theoretical underpinnings. As outlined by Vischer,^1^ deterministic perspectives that view user behaviour as a result of the environment are located at one end of the conceptual spectrum opposite social constructivist approaches which emphasise the importance of social context over the built environment in explaining behaviour. Over the last 20 years, the majority of empirical publications on design quality have tended to comply with the deterministic perspective, with typical studies seeking to identify how the design of the building environment impacts on the user producing outcomes in health and well-being, behaviour and performance. These studies, if not directly allied to, are often tacitly founded on the principles of environmental psychology or sensory neuroscience with an emerging research agenda around the holistic impact of design on the human senses.^4^ The significance of social context is notably absent from the majority of this literature.

### Social context in building design quality

Human relations are an unequivocal presence in occupied buildings. The relationship between building design and building users does not occur in a social vacuum, yet design quality evaluation rarely considers social context as a mediating factor. Buildings house a distinct milieu of users: people with individual identities structured, either formally or informally, into groups of various scales and engaged in dynamic sets of social relations.^1^ To illustrate, within any typical office building there exists a large number of employees, each with individual personality traits and behavioural characteristics. Based on the way that the organisation is run, employees are formally structured in groups, both spatially by workgroup (the people sharing a specific working area)^5^ and spatially or non-spatially by team and/or department (the people working together based on a project or task), as well as informally structured in groups based on social ties like collaboration or friendship, also spatially and non-spatially. Every building user is simultaneously a part of multiple building user groups at nested scales, including the individual user, the workgroup, team and/or departmental user, and the organisational user.^1^ Inherently linked to this social structuring are sets of ever-changing social relationships between building user groups at each scale. The picture becomes further complicated in the case of a shared, multi-organisational office building, or a more complex socio-spatial environment with multiple types of interacting user groups moving in and out of different spaces, for example, patients, visitors, doctors, nurses, administrative and maintenance staff in a hospital. The influence of building design quality on the people using it is irreducible from the social context of that environment. The outcomes of design that accrue to building users, whether they are health and well-being, behaviour or performance related, are unavoidably influenced by the social context that encapsulates them. The existence of fluid social relations within and between user groups at various scales is captured by the term ‘building user group dynamics’. It is defined as a combination of three interrelating scalar elements: (1) organisational cultures, referring to the traits and mores making up the social order of the organisational, or institutional, user,^6^ (2) management strategies, referring to the systems and processes that control/enable^7^ individual users and user groups, and (3) social norms and practices, referring to the tacit knowledge and related behaviour patterns of individual users.^5,8^

### Paper aim

There is an increasing need to understand the social context of buildings and building design in use, made apparent by the recent surge of interest in ‘social value’^9^ and its applicability to user-centric evaluation in post-occupancy building research.^10^ Promoting a nuanced understanding of building users in situ represents an opportunity for future design quality research. The aim of this paper is to address this gap, reviewing empirical research in the design quality field in relation to building users and the influence of building user group dynamics. The review provides an original in-depth study of the existing research across a typology of buildings to contribute to and advance current debates in studies of design quality.

### Methodology

This review is based upon the analysis of over 200 publications. It is not presented as an exhaustive review of all user-centric design quality evaluation research, which is great in number and considerably wide-ranging. It is an in-depth exploratory review to generate an improved understanding of how building users and their social context are addressed in the design quality literature. The identification of literature for the review was based on published, English language, peer-reviewed work in academic journals, academic conference proceedings and relevant public sector outlets. As this is an inherently inter-disciplinary field, specialist databases were omitted in favour of a wide-ranging search. A flexible approach was taken with various search terms to allow for a comprehensive exploration of the variety of research, academic and public, that addresses design quality issues. Key search terms used singularly or in combination included ‘design quality’, ‘building design’, ‘building users’ and ‘social value’, with searches for similar words or synonyms, for example, building user/occupant/resident, etc. In addition, the reference lists of publications identified in the literature search were examined for other relevant studies in an informal snow-balling technique.

The selection of publications for inclusion in the review was based on the following parameters:

- post-occupancy building research of user-centric design quality evaluation;
- empirical studies at the scale of the individual building as opposed to public realm or neighbourhood scale;
- conceptual or review publications addressing this field of research, and,
- research conducted in developed countries.

Publications that did not meet these criteria were excluded, for example, research relating to the design or construction stages of development as opposed to the evaluation of occupied buildings or that conducted in developing countries. A wide range of literature was selected for review including studies from building science, construction and engineering, facilities, human geography, clinical medicine and health studies, environmental psychology, business studies, retail studies as well as public sector outputs. The literature was analysed thematically according to the paper’s aim to understand design quality research in relation to building users and the influence of building user group dynamics. The themes under exploration were the user outcomes being studied, the handling of building users, and consideration of building user group dynamics. Analysis identified five building types that dominate the design quality literature and the review was therefore conducted across this typology, with the paper structured accordingly. Both commonalities and gaps in the literature were identified and located by building type to form a comprehensive picture of how research on building design in use has developed and can be applied to emerging challenges. The key findings have been presented visually in two tables. The first summarises the user outcomes that dominate each building type and the second identifies how the user is conceptualised in each building type.

**Table 1.** Common user outcomes by building type studied in the design quality literature

	Health and well-being outcomes	Behaviour outcomes	Performance outcomes
Housing	Clinical health,^25^ mental health,^37^ depression,^27^ thermal comfort,^28^ well-being,^24^ feelings of security and privacy^13^	–	–
Workplaces	Satisfaction,^44^ thermal comfort, acoustics, air quality, lighting and daylighting,^14^ feelings of privacy,^38^ sense of belonging^42^	Environmental control,^5^ territoriality and belonging,^19^ social interaction and collaboration,^42^ recruitment and retention^55^	Productivity^5,43^
Healthcare	Clinical health,^12^ satisfaction,^60,69,77^ well-being,^20,61^ indoor environmental quality,^63^ safety at work^17^	Environmental control,^60^ walking distances, recruitment and retention^17^	Quality of care^17,71^
Education	Satisfaction,^82^ thermal comfort,^108^ acoustics and lighting^83^	Student enrolment,^94^ attendance and absenteeism,^81^ staff recruitment and retention,^84^ customer footfall,^103^ re-patronage intentions^102^	Academic engagement and achievement,^15,18^ academic research output and collaboration^96^
Retail/service sector	Feelings of satisfaction^112^ and irritation,^119^ cognition and emotional response,^120^ pleasure-feeling,^109^ disconfirmation,^121^ perceived quality^118^	Approach-avoidance behaviours,^116^ consumer behaviours like buying and browsing,^16^ re-patronage intentions^122^	–

**Table 2.** Conceptualisation of the building user found in the design quality literature

	Housing	Workplaces	Healthcare	Education	Retail/service sector
Emphasis on measuring user outcomes					
Health and well-being	✓	✓	✓	✓	✓
Behaviour	✗	✓	✓	✓	✓
Performance	✗	✓	✓	✓	✗
Analysis of building users					
User identity	✗	✗	✗	✗	✗
Existence of building user groups	✗	✗	✓	✓	✗
Existence of building user group dynamics	✗	✗	✗	✗	✗
Consideration of building user groups					
Organisational cultures	✗	✓	✓	✗	✗
Management strategies	✗	✓	✓	✗	✓
Social norms and practices	✗	✓	✗	✓	✗

## The user in context in design quality research

The empirical work on design quality is united by its focus on the benefits (and dis-benefits) that accrue to the users of buildings which, being problematic to measure, have been termed ‘intangible outcomes’.^11^ The majority of the design quality literature addresses the impact of design by identifying and trying to capture a range of outcomes in the user related to three identifiable categories: health and well-being, behaviour and performance. Health and well-being outcomes concern how users feel, physically and mentally, and examples include recovery rates in hospitals,^12^ incidence of depression in social housing^13^ and satisfaction in office buildings.^14^ Behavioural outcomes relate to user actions, for example, the attendance rates of school children^15^ and consumer approach behaviours in retail environments.^16^ Performance-related outcomes are associated with user achievement of goals or targets, like productivity in office workers,^5^ the quality of care delivered by nurses^17^ and the academic achievement of students.^18^ However, explicit categorisation of user outcomes is rarely made in the design quality literature. Typically, a number of predetermined outcomes to be explored empirically are identified based on a discipline-specific framework or model.

In the relationship between design and building users, the influence of social context as a contributing factor is generally not addressed. Social context is conceptualised here in terms of building user group dynamics, entailing an amalgamation of organisational cultures, management strategies, and social norms and practices in the context of each building. There are an emerging number of studies that acknowledge the role of contextual factors in mediating the impact of design on the building user.^19–22^ However, they tend not to examine the make-up of different user groups or their dynamics in depth and there is little discernible consistency in how this is conceptualised or how its effect is measured. The following sections review the design quality literature across five key emergent building types: housing, workplaces, healthcare, education, and the retail/service sector, focusing on how the user is framed, the types of outcomes commonly investigated, and the extent to which user group dynamics are considered in the relationship between design and user outcomes.

### Housing

Design quality researchers have conducted numerous studies on housing, with contributions from disciplines including building science, health studies, sociology, environmental psychology and clinical medicine. A neighbourhood scale or urban design perspective is more common than research on individual buildings and the majority of the existing work addresses the physical condition of residential buildings, rather than their architectural design features. A common thread running through these studies is the association made between poor housing conditions and health (both self-reported and clinical conditions like respiratory disorders) and well-being outcomes.^23,24^ This includes a small literature on children’s respiratory health specifically,^25,26^ as well as studies on mental health and the incidence of depression in relation to well-being measures like security or status.^13,27^ Research on the health and well-being ‘co-benefits’^28^ of housing retrofit due to improved thermal comfort is also prominent,^29–31^ including the impact of retrofit on mental health^32^ and on elderly health.^33^ There is no research on wider notions of well-being, like inter-family relations and family breakdown, influenced either by housing conditions or residential design.

Overall, the majority of the research outcomes are health and well-being related, and behavioural outcomes represent a gap in design quality research with relevance to environmental behaviour change in homes (examples of common user outcomes under study in domestic buildings, as well as the other building types, can be found in Table 1). Furthermore, housing studies that address architectural design, rather than housing conditions, are generally missing. Weich et al.^34^ contribute a study on the links between mental health and depression and urban built environment design, rather than conditions, but this is at a neighbourhood scale. However, there is an emerging literature relating to the ‘building for life’ and ‘ageing in place’ ethos with an emphasis on flexible design for changing user requirements, as exemplified by the ‘Lifetime Homes’ design standards. This research area has links to elderly user experience and independence^35,36^ and mental health.^37^

There is scant attention paid to the role of social context in mediating the relationship between householders and their housing. Wells and Harris^13^ consider social withdrawal as a mediating factor in the association between housing quality and psychological distress in low-income women, where substandard housing restricts socialising within the home, creating the potential for reduced social networks and poor mental health. The notion of building user groups does not seem immediately applicable to typical single-family housing, however, maintenance and management by an external organisation play a role in a wide variety of housing types, including rented and social housing, private flats and apartments, assisted living and other dwellings that share external space with neighbours. The nature of this external influence, the speed of response to requests and typical level of interaction with dwellers is anticipated to have an important effect on the experience and behaviour of the user in that environment. In addition, the impact of different domestic management preferences and styles on how the people living in that space enjoy its design is also assumed to be a significant variable. This represents a currently unexplored area of research into design quality which would feed directly into the identified gap on inter-family relations. It is also noted that housing studies tend to focus on nuclear families and elderly residents, with little consideration of other living situations.

### Workplaces

Workplaces are the subject of a well-developed empirical literature on design and users which is dominated by research on commercial office buildings, both conventional and green. There is a remarkable gap in design quality research addressing workplace environments and users beyond office buildings. For example, factories, workshops and studios are not represented in these studies. The existing array of office-based studies display a distinctly commercial nature, focusing on individual productivity and the impact of architectural design, interior office layout, indoor environmental quality and aesthetics.^5,38,39^ A wide range of user outcomes are addressed, covering health and well-being, behaviour and performance related categories. Office user satisfaction with the working environment is a common research topic, involving thermal comfort, lighting, acoustics, and feelings of privacy and security resulting from the layout of office furniture.^14,40^ This tends to be linked to environmental control behaviours,^41^ territoriality and social interaction,^42^ and individual productivity.^43^ Typically, the collected data is self-reported and is justified as an appropriate people-centred method to reveal end-user opinions.^43^ However, the validity of self-reported feedback is questioned by others.^1^

Whilst satisfaction is one of the most common health and well-being outcomes found in the workplace literature, it is rarely studied contextually with reference to the mediating effect of user group dynamics. For example, few studies address the importance of progressive management and communication strategies for the attainment of user satisfaction with their environment. A study by Kato et al.^44^ describes the importance of small-scale issues to users, such as personal comfort, which require greater attention from management when moving into new buildings. The findings evidence the success of management strategies that educate users to read tenant guides and optimise their personal working space, and create opportunities for two-way communication between users and management.

The influence of user group dynamics on the behavioural outcomes of individual users is also seldom addressed. For Vischer,^45^ corporate structure and culture act as significant variables in the relationship between environment and user. She argues that a flat organisational structure with an egalitarian culture encouraging decentralised decision-making promotes very different user interaction with design than a hierarchical firm with a competitive and disciplinary culture. Unpacking corporate culture further, managerial and operational decisions are argued to have a significant effect on user behaviour. The influence of managerial culture on users can be categorised into two aspects: normative (how users think they should interact with design) and perceived (how users actually interact with design). The former is addressed by Wells et al.^19^ in their study on workspace personalisation. They find that organisational policies and norms play a more influential role than personal factors on employee interaction with their personal environment, evidencing the inclusion of corporate cultures as a mediator of design/user interactions. The latter refers to slow and unresponsive facilities management cultures and the knock-on effect on user annoyance with design, which can cause users to bypass certain systems in an overcompensated reaction.^46^ In this way, behavioural user outcomes are mediated by a combination of normative and perceived dynamics within different organisational user groups. A related literature aims to model user control of the office environment through quantification and digital modelling of observed window use.^41,47,48^ This research is typically undertaken in green office buildings and is linked to user comfort and control in the promotion of building performance, rather than user performance.

As discussed, individual productivity within the office environment is the predominant performance-based outcome under study. In relation, acknowledgement of user group dynamics as an important factor is much more prevalent in studies focusing on productivity rather than well-being or behaviour-based outcomes. Office norms or practices, organisational culture and management are all tackled in this respect.^7,8,49^ In addition to physical design and layout, individual productivity is argued to be affected by the ‘behavioural environment’ of the office, a set of dynamic elements like interaction and distraction,^50–52^ the product of a variety of contextual factors such as organisational purpose and nature of work.^38^ As a result, the ‘connectivity’ of the office layout is theorised to impact on social interaction and innovation opportunities, affecting productive outputs.^53,54^ In addition, organisational norms and working practices also dictate workgroup size with implications for environmental control, personal comfort and associated productivity gains and losses.^5^ However, commercial practice has yet to understand the workplace environment as an asset in need of strategic management in order to enhance productivity and improve organisational performance.^55,56^

There is a concentration in the commercial office literature on the measurement of individual productivity outcomes in relation to design, and this has also produced the most developed research into the role of building user group dynamics. In contrast, a separate area of design quality research uses business performance and organisational outputs to evaluate office design.^56,57^

It is important to note that the majority of design quality research in offices treats the user in a broad sense without making distinctions between different groups of users, for example, based on management grade or activity. In comparison, commercial post-occupancy evaluations do treat management grade as a significant factor in user experience of the office. Understanding more about the ever-changing social relations that exist between user groups and the mediating impact of this on users’ perceptions, usage and performance within an office environment represents a less well-studied element of the social context in workplaces.

### Healthcare

Design quality research has often focused on healthcare buildings. This is an unsurprising trend considering the environments required for health and well-being of patients that represent the primary function of these facilities. In particular, hospitals form a key literature addressing user experience from the perspective of both patients and healthcare professionals. In the former (and more extensive) literature, clinical outcomes-driven design has steadily given way to conceptualising the patient as a customer in the discourse of ‘therapeutic environments’.^58^ Health and well-being outcomes are prioritised and typical examples include clinical health improvements, length of stay, satisfaction, environmental control, feelings of territoriality and privacy, access to outdoor spaces and ease of wayfinding.^12,59–64^ The impact of design on aspects of patients’ well-being like dignity, autonomy and empowerment is also common, especially in mental health facilities research.^65,66^ There is also a subset of studies that addresses the impact of design on paediatric patients specifically.^67–69^ Aside from environmental control, behavioural and performance-based patient outcomes (synonymous with healing and well-being in a healthcare environment) are not addressed in the literature.

The second area of research addresses the impact of hospital and ward design on the healthcare professionals working in the space. Whilst less developed than the patient literature, it comprises a wider range of outcomes. This includes satisfaction, indoor environmental quality and safety at work,^69,70^ walking distances, recruitment and retention,^17^ and the quality of care delivered,^71^ signifying academic interest in health and well-being, and behaviour and performance related outcomes. It is predominantly nursing staff who feature in these studies^72^; other hospital workers, including doctors, pharmacists, technicians, administrative and maintenance staff are rarely included in healthcare design quality work (although for a consultant perspective, see Curtis et al.^65^).

Although the user perspectives of patients and healthcare professionals have been dealt with separately in this review, many of the empirical studies discuss user outcomes from patients and staff in combination. It is noted that the research field does not address the complex social relations that exist between various user groups and their mutual impact on each other’s experience of the hospital environment. For example, Whitehead et al.^64^ suggest that perceptions of cleanliness have a significant impact on patient satisfaction with their stay, and the maintenance staff group is evidently implicated in this process. In addition, a third user perspective can be identified based on the impact of design on hospital visitors, including the parents and guardians of paediatric or vulnerable patients.^61,69^ However, the low number of existing studies suggests that this is under-researched.

A striking characteristic of the healthcare sector design quality literature is the dominance of inpatient environments of hospitals and a lack of studies on outpatient or primary care facilities, such as surgeries, clinics and support centres (see Raleigh et al.^73^ for a report on GP surgery facilities). Within the inpatient studies on hospitals, there is a distinction between research that addresses hospital buildings in general, studies on particular hospital types, like paediatric^68^ or geriatric hospitals,^74^ and studies which select a specific environment, for example, wards^71^ or patient rooms,^60^ or a specific department, for example, orthopaedic units.^75^ In line with the lack of research on visitor perspectives, there are no existing studies on day rooms or waiting areas. In addition, user group dynamics are not studied within this literature, in relation to working practices, organisational culture or management strategies.

There is some developing work in non-clinical healthcare environments. Care homes for the elderly and the role of tele-care have been studied with health and well-being outcomes of inhabitants and satisfaction outcomes of staff prioritised.^20,76^ Furthermore, within this group of studies, there is evident reference to the significance of user group dynamics. Torrington^20^ outlines the complex interaction between building design, elderly users, care staff and building managers which has a direct impact on patients’ well-being. Corporate cultures that prioritise safety significantly reduce opportunities for patients’ environmental control and pleasurable activities, directly impacting on the quality of life they can enjoy. This sort of cautious management culture is typically found in new, highly regulated care homes that meet stringent design standards, including restricted outdoor access, heavy fire doors, and featureless and confusing circulation routes, all of which limit freedom of movement and choice of activity.^20^ In relation, Parker et al.^77^ found improved staff morale in non-institutional environments. This simultaneously evidences their protective nature towards patients, yet is partly responsible for the culture of over-regulation and risk minimisation that has emerged. An audio recording on the Design Council website of a presentation by an architect specialising in care homes for elderly dementia sufferers reiterates the significance of managerial culture on design and user interaction. His experience of a small-scale facility with amateur, co-produced care of patients taken over by care professionals led to the removal of the social hub to create a central nurses’ station for access and surveillance purposes.^78^

Other public sector research continues to look beyond hospital environments, with a recent study addressing user experience of design in an outpatient pharmacy, including patient and staff perspectives.^79^ An online magazine article addresses the related issue of empowering design in shelters for domestic violence victims and recognises both inhabitant well-being and staff morale,^80^ highlighting the design of welfare-related buildings like shelters and children’s homes as an important research gap.

To summarise, whilst the healthcare building literature is significantly dominated by design quality research on hospitals from both a patient and a staff perspective, there is little analysis of how the various user groups within the environment interact and produce changing sets of social relations to structure user interaction with design. In contrast, the relatively recent emergence of care homes as a source of academic attention has focused on the role that managerial cultures play in patient and staff experiences, highlighting the relevance of user group dynamics as a variable. Substantial research gaps exist on outpatient or primary care building types, as well as the perspective of visitors within the social spaces of healthcare environments.

### Education

Educational buildings represent a significant proportion of the research on building design and its impact on building users, with the majority of literature being divided between schools and universities. Related to these building types are libraries and historic buildings, which are covered briefly at the end of this section. The wide-ranging empirical research on school buildings can be distinctly split between two user perspectives, student and teacher, and tends to evaluate design against a variety of user outcomes, including health and well-being, behaviour and performance. Examples include satisfaction^81,82^ and the impact of acoustics, lighting and thermal comfort,^83^ student attendance/absenteeism and staff recruitment/retention,^15,84^ and academic engagement and learning outcomes.^18,85,86^ It is noted that school design tends to be evaluated based on traditional ‘chalk and talk’ teaching rather than new ‘effective learning environments’.^11^ The majority of research focus on student well-being, behaviour and performance outcomes, whereas clinical health outcomes are less relevant in this sector. Whilst the impact of school design on teachers’ well-being and behaviour is tackled to some degree,^87^ this represents a research gap in the design quality literature, linked to the absence of extensive workplace research beyond offices. Both primary/elementary and secondary/high school buildings are included in empirical research on the impact of design.

There are several school studies where the concept of user group dynamics is related to performance-based user outcomes. The significance of what is termed ‘school climate’ in mediating the relationship between facilities quality and academic achievement is identified statistically.^21^ School climate represents contextual products of poor facilities, such as reduced morale, engagement and effort of the school community, found to act as a variable in the achievement of learning outcomes. In a follow-on study, ‘learning climate’ is conceptualised as the interaction of intended design, the day-to-day-realities of design, and the occupants.^88^ The learning climate is understood to facilitate or limit environmental understanding and control with implications for effective academic learning. In relation, a separate study advocates that building purpose (i.e. educational function) should be taken into account when investigating the relationship between school facilities and academic achievement,^89^ re-emphasising the significance of social context in understanding design/user interactions.

Although noticeably smaller than the literature on schools, existing design quality research on higher education buildings and universities in particular has two main themes: the impact of design on users with respect to learning and teaching outcomes and research outcomes.^90^ An underlying commonality across studies addressing learning and teaching is the role of information and communication technologies as a driver for change in the sector, with discussion of flexible learning environments and other pedagogical issues like group learning and mobile learning.^91,92^ The user outcomes related to learning and teaching are similar to those in schools, comprising student satisfaction (well-being), student enrolment and attendance (behaviour), and student academic achievement and learning (performance).^93–95^ However, the design quality work on learning environments in universities tends to be relatively discursive with a lack of empirical evidence. Furthermore, there is no research on these spaces from the lecturer perspective.

The impact of building design on academic research outputs is a newly emerging design quality literature which displays similarities to the publications on office buildings. The focus on users in academic workspaces relates to the impact of various office types on academic output and collaboration.^96^ Significantly, many of these studies emphasise the role of user group dynamics in mediating the impact of design on well-being outcomes. Institutional management is argued to influence academics’ personal control over space and perceived embodiment of respect in the working environment, directly influencing feelings of satisfaction, autonomy and worth.^97,98^ In relation, Pinder et al.^96^ discuss how institutional norms affect researcher expectations due to prior experience. Changing space provisions, typically from allocated desk spaces to non-territorial hot-desking, are usually accompanied by low satisfaction outcomes from users with previously allocated desk spaces compared to higher satisfaction from users previously without desks.

In relation to the work on schools and universities, the design of library buildings and its impact on library users represents a significant although less extensive literature, on both academic and public libraries. The former area of research tackles similar technological drivers for change as in university buildings work, namely, a technology-literate generation of students with shifting user requirements producing a trend for value-adding elements such as social learning commons in the ‘library as place’ debate.^99–101^ Again the focus is primarily on student well-being outcomes, such as satisfaction.

Design quality work on public libraries is less common than academic libraries, although there is a similar focus on well-being outcomes, such as user satisfaction. In addition, an association with the service sector literature (discussed below) is identifiable, with behaviour-related outcomes being prioritised.^102^ Conceptualising library users as customers leads to the prevalence of well-being outcomes for the attainment of desired behavioural outcomes, such as user footfall.^103^ This questions the core purpose of libraries, producing a tension in their design and intended use between grand and ‘seductive’ architecture, legitimised by the attraction of regional tourist users, and small-scale neighbourhood design which prioritises the local community user.^104^

Finally, a small spinoff in the educational design quality literature addresses the user in relation to historic building design. Whilst user experience is implicit in research on ‘built heritage’ and heritage tourism,^105^ there is minimal discussion of the impact of historic building design on user outcomes. However, a niche literature on European churches exists which relates user well-being to improvements to thermal comfort made possible through innovative technologies.^106–108^

The wide range of buildings that fall into the education typology have spawned a variety of design quality studies that do not necessarily share conceptual or methodological ideas. The schools literature is primarily empirical with a focus on student well-being, and behaviour and performance related outcomes. There are increasing references made to the significance of user group dynamics, such as school or learning climate, as a contextualising variable in design/user interactions. In contrast, the university literature is much less empirically developed and tends to discursively outline the drivers for change and how this can be facilitated through design, rather than analysing that design in use. There is an overall trend towards the student perspective across both sectors, whilst teachers and lecturers are considerably understudied, linking back to the lack of workplaces research beyond offices. The addition of building types such as libraries and historical churches further divides this field by conceptualising users as customers and focusing on well-being outcomes to encourage continuing use, echoing service sector research.

### Retail/service sector

A substantial component of the literature on design and users is comprised of the wide-ranging retail and service sector, including clothing shops,^109^ supermarkets and grocery stores,^110^ restaurants,^111^ banking services,^112^ hotels,^113^ sports venues^114^ and museums.^115^ Typically, retail and ‘servicescapes’ research does not investigate the impact of architectural design on the user; rather, the sophisticated concept of ‘atmospherics’ dominates the literature, referring to the holistic use of various environmental cues and stimuli to influence users, for example, layout, lighting, music and scent.^16,116,117^ The impact of atmospherics captured in the literature falls mainly into two categories: well-being outcomes, such as feelings of satisfaction/irritation, cognition and emotional response, pleasure-feeling, disconfirmation (fulfilling expectations or not), and perceived quality,^118–121^ and behavioural outcomes, such as approach-avoidance, consumer behaviours such as buying and browsing, and re-patronage intentions.^16,109,111,120^ The majority of studies address both well-being and behavioural outcomes in customers, often investigating a causal link between cognitive responses and related consumer behaviours. Notably, the user outcomes under study differ significantly from those in other building types due to the distinctly commercial purpose of the retail and service environment.

The emphasis on the customer perspective is ubiquitous and there are no studies that focus on the retail and service sector staff that work in these environments. However, user group dynamics are acknowledged in several building types, especially in relation to the influence of management. The concept of human factors has emerged in the servicescape literature to describe how management elements have a direct influence on customer experience and perception of service quality. There is an emerging interest in the interaction of different user groups within these spaces, although the preoccupation with the impact on the customer remains. For example, in restaurant research, Harris and Ezeh^22^ have employed the term ‘social-servicescape’ to refer to the management of the establishment, customer service and staff image, which influences customer experience, disconfirmation and interaction with the environment.^121,122^ The significance of human factors is also addressed in design quality research in hotels where the role of staff manner and image is understood as a variable in visitor experience of hotel design.^123^ A related concept in the hotels literature is ‘ambience’, a similar idea to retail atmospherics. Heide et al.^113^ discuss the significance of hotel management in creating and producing an ambient atmosphere, a subjective phenomenon unique to each hotel, directly influencing how guests respond to the environment.

Design quality research in this sector predominantly addresses shops, restaurants, hotels and banks, whereas entertainment venues, such as stadia, theatres and nightclubs are less well studied. There is a focus on measuring user outcomes in the customer, whereas staff perspectives are entirely ignored. In addition, the influence of architectural quality is wholly missing from retail sector studies, and represents a considerable research gap. Due to the purpose of the retail and service environment, performance-related outcomes are not studied, whereas well-being and behaviour-related outcomes are studied in tandem. There is an emerging interest in the impact of human factors on customer experience, including management cultures, that represents an awareness of building user group dynamics as a variable in the relationship between users and design.

A relevant but niche research area is the design of transport-related buildings, such as airport terminals and railway stations. The limited literature mainly focuses on airports in terms of wayfinding design and its impact on user satisfaction and the perceived ‘level of service’^124^ with typical outcomes including cleanliness, lighting and walkability.^125^

## Discussion

This in-depth review of the design quality literature has generated a more comprehensive picture of this empirical research area, including its interpretation of the building user and the concept of social context (as illustrated by Table 2). The treatment of the building user is distinct with an emphasis on identifying and measuring user outcomes. In contrast, the social context that surrounds the interaction between design and users is generally neglected. Design quality research has focused on certain building types, particularly commercial buildings, such as offices and retail, and important public sector buildings, such as hospitals and schools. In addition, there are some emerging areas of interest that overlap between building types, for example, the burgeoning work on care homes draws on previous work from both the domestic and healthcare spheres.

Some significant research gaps are also evident. Housing is primarily evaluated based on its condition rather than architectural design and there is no work on the impact of design on inter-family relations or alternative household situations. Workplaces other than offices are considerably under-represented in the design quality literature. Out-patient surgeries, clinics and support centres form a similar gap in the healthcare sector that is dominated by work on hospitals. Within the hospitals literature, patients are most commonly addressed whereas healthcare staff (other than nurses) and visitors are rarely studied, and the social areas of hospitals (day rooms, waiting areas) are not considered. Related buildings, such as shelters and children’s homes, are wholly absent from design quality research. The perspectives of teachers and lecturers in educational buildings are less-commonly studied than those of students, and empirical design quality research in universities is relatively sparse. Other educational design quality work on libraries and historic buildings is also lacking. There is little research on the architectural design of retail and service sector buildings, whilst the existing work on atmospherics does not address entertainment venues to the same extent as shops, restaurants and banking facilities. There is no research on the user outcomes of staff working in retail and service sector buildings.

A significant finding from the review has been the inconsistency across building types with respect to user outcomes. This is assumed to be a result of the discipline-specific nature of design quality research: empirical activity tends to focus on a particular building type without drawing on academic developments made in relation to the study of other buildings. Therefore, there is a notably wide range of user outcomes scattered across the different types of buildings in the design quality literature. However, once the variety of user outcomes is ordered into the three categories identified here (health and well-being, behaviour, and performance), trends begin to emerge, as Table 1 highlights. These patterns are a function of the building type under study, where building purpose directs the measurement of user outcomes relevant to that environment. The domestic literature tackles predominantly health and well-being outcomes, as would be expected in buildings that provide living environments where people spend substantial amounts of time and cannot easily leave substandard spaces or simply swap them for an improved one. Performance outcomes are not relevant in this sort of building as users are not involved in the production or achievement of an end goal, whilst behavioural outcomes have been identified as a significant gap in research.

As presented in Table 1, the workplaces, healthcare and educational building research areas address the full range of health and well-being, behaviour and performance user outcomes. Users of these environments are expected to spend considerable amounts of time within them and have relatively low levels of autonomy in terms of improving or leaving the environment, making health and well-being outcomes particularly relevant. Behavioural outcomes are pertinent in buildings that represent public environments with a diversity of users, to understand the user activity being accommodated as well as promoting desired behaviours in these spaces. These building types also have work or education functions, generating an emphasis on performance outcomes, specifically how design influences individual productivity, quality of care and educational attainment.

The retail and servicescape literature deals with the first two categories of well-being and behaviour, but there is a noticeably commercial emphasis on the sorts of outcomes. For example, well-being outcomes relate to customer satisfaction and experience, and behavioural outcomes are related to consumer behaviours and actions. This is in contrast to the outcomes studied across the other building types in terms of health and well-being, also illustrated in Table 1. In addition, performance outcomes do not feature in this typology as the overwhelming customer focus has overshadowed staff performance. Overall, Table 1 demonstrates that after the outcomes under study are categorised, trends begin to emerge between building types that are otherwise less visible. Understanding where similar user outcomes are of interest in other disciplinary research areas facilitates the potential cross-over of ideas between previously unconnected studies. For example, the wealth of empirical research conducted in office buildings represents a valuable resource in emerging discussions about changing trends in academic workspace, whilst the sophisticated ‘atmospherics’ frameworks employed in retail and service sector work are applicable to academic and public library debates.

Whilst the variety of user outcomes under investigation appears relatively disjointed on first inspection, the treatment of the building user in design quality research is highly distinctive, as summarised in Table 2. There is a preoccupation with user outcomes rather than analysis of the users themselves. Across the various building types, empirical studies favour the anonymous user with few defining features. For example, studies on housing, offices, retail and the service sector typically address the householder, office worker and customer, respectively. Some building types are more likely to be studied with an acknowledgement that different user groups exist, for example, patients, visitors and healthcare staff in hospitals, students and teachers in schools, and students, lecturers and academic researchers in universities. However, the implication of multiple user types is not investigated and one or two user groups tend to dominate in each building type, for example, the patients in hospitals or the students in schools.

Consideration of building user group dynamics as a contextual factor in the relationship between design and user is rare. The building types identified in Table 2 comprise workplaces, specifically offices, healthcare sector care homes, schools and academic workspaces in the education typology, and the service sector. However, there is no defining characteristic or framework in how user group dynamics are conceptualised or handled in these studies, with considerable variety in the contextual elements identified as mediating influences. This can be explained partially by the discipline-specific nature of the vocabulary found in each area of research, combined with limited sharing of ideas between disciplines. Furthermore, there is variation in how user group dynamics are perceived to mediate the interactions of design and users. This is related to the focus on outcomes in a single user group in office, academic workspace and service sector research, whereas work in care homes and schools recognises the presence of multiple user groups, also outlined in Table 2.

The existing design quality studies that address building user group dynamics are few in number, making the identification of patterns or trends problematic. However, three main components recur in the literature, relating to organisational cultures, management strategies, and social norms and practices. Building user group dynamics represent a significant research gap across the whole design quality literature and provide an opportunity for empirical attention in the future.

Recent public sector interest in valuing the design quality of the built environment^10,126^ has not included an appreciation of social context or, more specifically, the potential of building user group dynamics to mediate the design/user relationship. Mulgan et al.^10^ consider multi-criteria analysis (MCA) methods, such as the Design Quality Indicator (DQI),^127^ to be the most common research technique currently used to evaluate design quality. The industrial post-occupancy evaluation, such as the Post-Occupancy Review of Buildings and their Engineering (Probe) studies,^128^ also falls into the MCA category of weighting and scoring systems. Common considerations of this family of tools can be categorised between the practical, such as functionality/usefulness, build quality and energy use, and the user-centred, such as occupant satisfaction.^129^ However, the evaluation criteria lack any reference to contextual factors, such as building user group dynamics, or their mediating influence on design quality.

Defining a framework for future investigation into the impact of building user group dynamics on the relationship between building design and users would drive a robust and coherent literature, rather than a disparate set of studies. This might entail the identification of key building user group dynamics and their categorisation into organisational cultures, management strategies, or social norms and practices. Analysis of their mediating influence on the existence and frequency of typical user outcomes for each building type would promote a more sophisticated understanding of how design, users and social context interact in the built environment. Further, it would facilitate development of new or amended design quality assessment tools that consider mediation by building user group dynamics as a critical element of the design/user relationship.

## Conclusion

This paper has summarised the design quality literature regarding the social context of buildings in use, reviewing the empirical research with respect to two cross-cutting themes: the measurement of outcomes for building users, and conceptualisation of users themselves. The typological approach has highlighted the relatively isolated academic developments in each building type that has produced a patchwork field of research, primarily unified by the dominant concern with user outcomes. The disinclination towards cross-disciplinary sharing has led to the study of a wide range of user outcomes. Emerging interest in the dynamics that exist between building user groups is developing in a similarly disparate fashion. There is significant potential for theoretical and empirical development based on the wealth of research available on various building types, but this opportunity is currently under-exploited due to a lack of interdisciplinary collaboration.

The importance of social context as a mediator of the relationship between building design and building users is yet to be fully explored. Developing a more nuanced understanding of building users in situ is proposed as an important opportunity for future design quality research. This would benefit both academic research and built environment professions by promoting environments that are designed for a dynamic community of building users rather than a set of anonymous and autonomous building user individuals. To develop an appreciation of the social relations that exist between people in buildings suggests that a wide range of perspectives would be of value to this endeavour, including a variety of social scientists as well as building scientists and environmental psychologists. For example, public spaces of non-clinical functions within healthcare buildings represent an under-researched area with considerable relevance. Corridor and waiting room environments are a routine part of user experience in primary care and hospital facilities, whilst increasing numbers of information and support hubs represent a new type of space in this sector. It is recognised that a variety of different user groups including patients, visitors, healthcare professionals and maintenance staff interact in such spaces, but the impact of these complex social relations on people’s experience and usage of the built environment is unknown. Pushing current research boundaries means going beyond recognition of different building user groups to include analysis of their social dynamics as an essential part of the social value agenda within buildings.
